# Cleansing efficacy of the electric toothbrush Oral-B^®^ iO™ compared to conventional oscillating-rotating technology: a randomized-controlled study

**DOI:** 10.1007/s00784-024-05882-1

**Published:** 2024-08-21

**Authors:** Anna-Lena Polak, Vera Wiesmüller, Lukas Sigwart, Nina Nemec, Lisa Niederegger, Ines Kapferer-Seebacher

**Affiliations:** University Hospital for Conservative Dentistry and Periodontology, Medical University of Innsbruck, Anichstr. 35, Innsbruck, 6020 Austria

**Keywords:** Powered toothbrush, Biofilm, Oral hygiene, Plaque reduction

## Abstract

**Objectives:**

This study aimed to compare the cleansing efficacy of the Oral-B^®^ iO™ electric toothbrush incorporating oscillating-rotating technology with microvibrations - with a traditional oscillating-rotating toothbrush.

**Materials and methods:**

Thirty adult participants were randomly assigned to use the iO™ electric toothbrush with the brush head iO™ Ultimate Clean or the traditional oscillating-rotating toothbrush Oral-B^®^ Genius^®^ with the Cross-Action brush head. Oral hygiene indices (Rustogi Modified Navy Plaque Index and Gingival Bleeding Index) were assessed before and after 28 days of home use of the assigned product. Participants were instructed to refrain from interdental hygiene during the study period. After a 2-week washout period, the clinical investigation was repeated in a crossover design.

**Results:**

All 30 participants completed the study with no dropouts. After 28 days of use, the iO™ showed statistically significantly lower plaque levels than the conventional oscillating-rotating toothbrush (25.09% vs. 30.60%, *p* = 0.029). This difference was particularly noticeable in marginal and approximal areas. There were no significant distinctions in gingival bleeding indices.

**Conclusions:**

The Oral-B^®^ iO™ electric toothbrush displayed enhanced plaque removal efficiency compared to a conventional oscillating-rotating technology.

**Clinical relevance:**

This study highlights the potential benefits of advanced toothbrush technologies for plaque reduction and encourages further research.

## Introduction

Nowadays, various manual and powered toothbrushes and brush head designs are available in the market. The industry aims to develop new and advanced technologies that maximize plaque removal and improve oral health. Powered toothbrushes have become increasingly popular due to their ease of use, highly effective plaque removal, and positive impact on oral health. Many advanced toothbrush models have incorporated bristle head designs, such as multi-level, criss-cross arrangements and rounded ends, to enhance plaque removal further. Several systematic reviews and meta-analyses have shown that powered toothbrushes are more effective than manual toothbrushes in reducing plaque and gingivitis in both the short and long term [1–3].

**Fig. 1:**
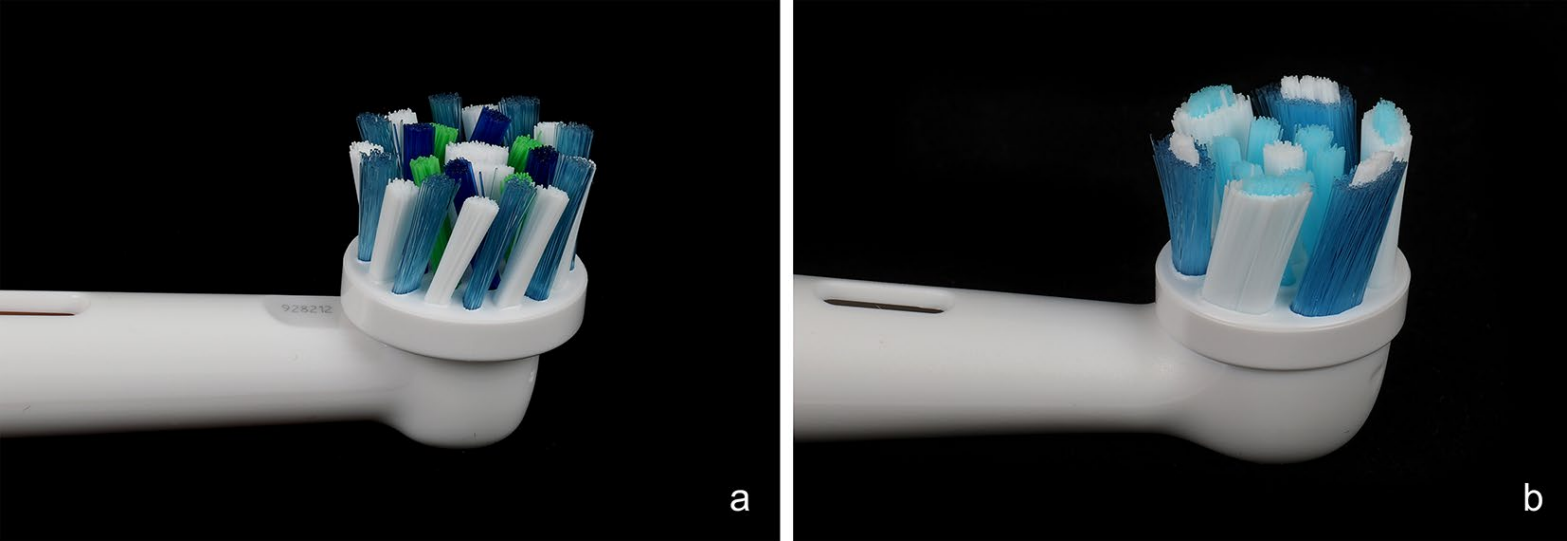
Brush heads of oscillating-rotating toothbrushes used in the present study. **(a)** The control product was the Oral-B^®^ Cross Action brush head with angled bristles. The bristle tufts of the two outer tings are alternately of different lengths with a height difference of 1 mm. **(b)** The test product was the “Oral-B^®^ iO™ Ultimate Clean toothbrush head” with a 2 mm increased diameter and Criss Cross bristles. The bristle field is slightly concave, sloping toward the center

Oscillating–rotating technology (O–R) with a round brush head was introduced by Oral-B^®^ in 1990. Over the past few decades, the size and design have been modified several times to improve the cleansing efficacy, patient compliance, and brushing experience. The Oral-B^®^ Cross-Action brush head with angled bristles has been the leading O-R head in various of brush series (Fig. 1a). Alongside the angled bristles, the head has micro-pulse bristles designed to eliminate interdental plaque. In 2020, Oral-B^®^ launched the new iO™ series that merges the oscillation-rotation mechanism with microvibrations produced at the site of plaque removal [4]. The handle of iO™ toothbrushes features a linear magnetic drive, which differs from the gear-based motor used in older O–R brushes. According to the manufacturer, this design reduces intrinsic energy loss and directs more energy toward the bristles [5]. It is compatible with a new set of round brush heads with an increased diameter 2 mm larger compared to the Cross-Action brush head and the Criss Cross bristles have a tuft-in-tuft design to ease interdental cleansing efficiency (Fig. 1b) [4]. Due to the brush head sizes, swiveling the brush head into the interdental spaces is no longer possible and is not recommended. Thus, the authors hypothesized that the larger brush head may clean the interdental spaces less effectively.

The company’s two clinical trials revealed that the Oral-B^®^ iO™ removed more plaque and significantly reduced gingivitis compared to a manual toothbrush [6]. Further manufacturer-funded research showed that the iO™ brush removes plaque more effectively than a sonic brush [7, 8]. Additionally, a significantly higher proportion of participants in the iO™ brush group progressed from “not healthy” to “healthy” gingival status within 24 weeks compared to the sonic brush group (96.4% versus 81.8%; *p* = 0.029) [8].

To date, there are no independent academic studies evaluating the iO™ technology, nor have any clinical studies been conducted to compare the iO™ technology with existing O–R brushes or brush heads. Thus, we aimed to compare the cleansing efficacy of two brush heads with different brushing technologies, the Oral-B^®^ iO™ Ultimate Clean brush head and the Cross-Action brush head with the previous O–R technology. The null hypothesis posits no significant differences regarding full-mouth plaque indices between the two types of toothbrushes and brush heads.

## Materials and methods

This study was ethically reviewed by the Ethics Committee of the Medical University of Innsbruck (EK Nr: 1379/2021). It was conducted per the Helsinki Declaration of 1964 and its later amendments. Before participation, all participants were given detailed information about the study’s conduct and provided written informed consent, including their consent to participate in this study.

### Study participants

Thirty adult volunteers were recruited at the University Hospital for Conservative Dentistry and Periodontology, Medical University of Innsbruck, from January 05 to April 01, 2022. Inclusion criteria were age ≥ 18 years, contractual capability, home use of an O–R toothbrush, and more than five teeth per quadrant. Exclusion criteria were a dental or medical profession or education, community periodontal index of treatment needs (CPITN) grade 3 or 4 [9], pregnancy or breastfeeding, systemic diseases or conditions that are associated with an increased risk of infection or necessitate concomitant antibiotic therapy with dental treatment, existing caries lesions requiring treatment, dental implants, and mental and behavioral disorders that impede (verbal) communication. Teeth with direct or indirect restorations were not excluded.

### Clinical intervention

**Fig. 2:**
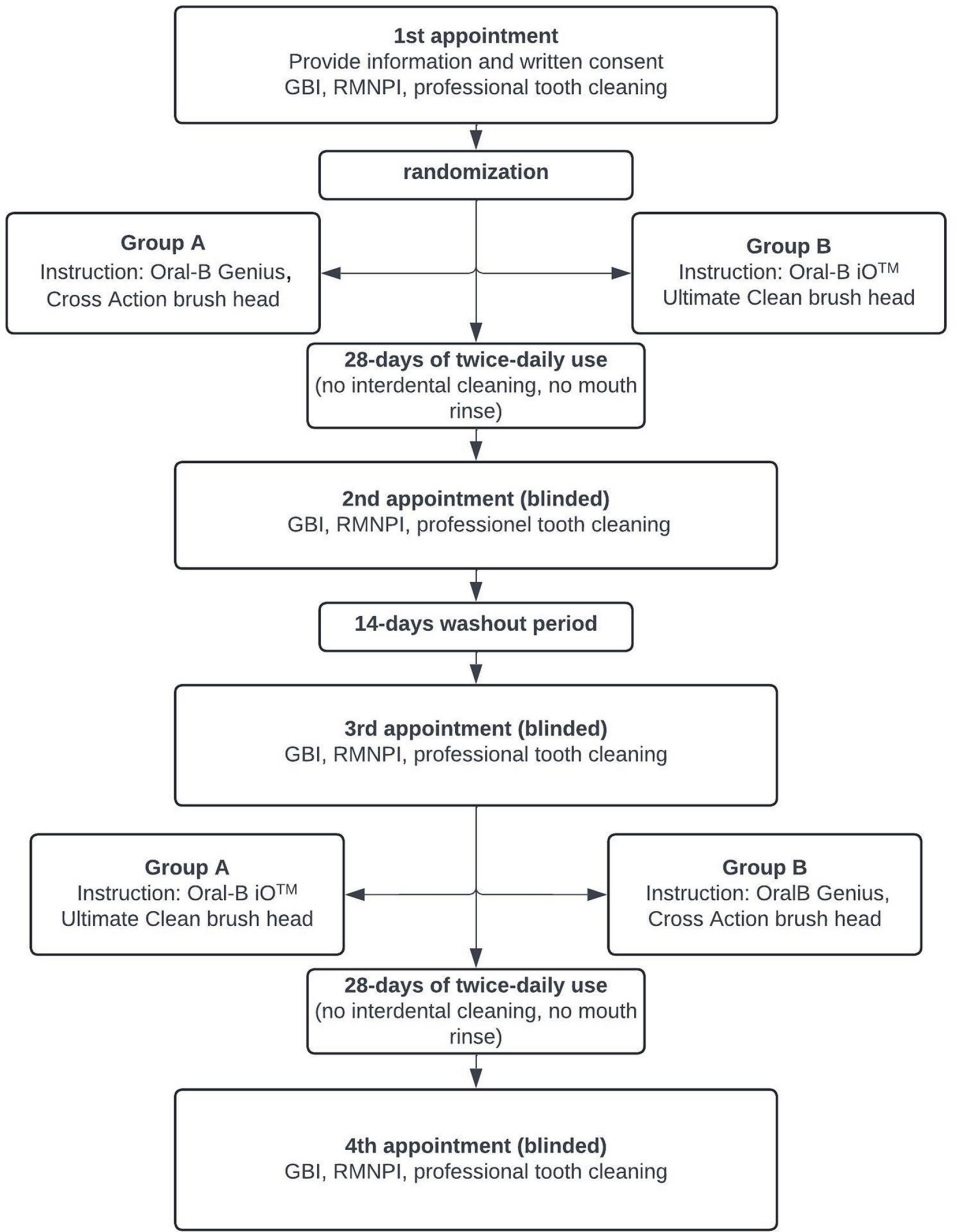
Visual outline of the study design. Thirty adult participants were randomly assigned to use the iO™ electric toothbrush with the brush head iO™ Ultimate Clean or the traditional oscillating-rotating toothbrush Oral-B^®^ Genius^®^ with the Cross-Action brush head in a crossover design. Oral hygiene indices (Rustogi Modified Navy Plaque Index (RMNPI) and Gingival Bleeding Index (GBI)) were assessed before and after 28 days of home use of the assigned product. Participants were instructed to refrain from interdental hygiene and the use of any chemical rinsing solution during the study period. After a 2-week washout period, the clinical investigation was repeated with the other product

This randomized-controlled, examiner-blinded, crossover study evaluated the cleansing efficacy of toothbrushing with the Oral-B^®^ iO™ Ultimate Clean brush head versus Oral-B^®^ Genius^®^ with the Cross-Action brush head (Figs. 1 and 2). Data collection was carried out from April 05 to July 30, 2022. The study design consisted of four appointments for each study participant. At the initial consultation, the participants received comprehensive information regarding the research protocol. They provided written informed consent was obtained before the inclusion and exclusion criteria were assessed. Study participants received the necessary toothbrushing products. Each cleaning cycle began with a new brush head.

Baseline data included the Rustogi Modified Navy Plaque Index (RMNPI) [10] after plaque disclosing with *2Tone* (Young, Earth City, Mo, USA), and the gingival bleeding index (GBI; Ainamo and Bay) [11]. The Rustogi Modified Navy Plaque Index (RMNPI) divides each buccal and lingual tooth surface into nine sections (A–I) to indicate the presence or absence of plaque dichotomously. RMNPI is the percentage of biofilm adhering sites to measured sites. It enables differentiation between the marginal areas of the teeth (A–C), interdental areas (D–F), or overall tooth surfaces (A–I). To evaluate gingival inflammation by GBI, a periodontal probe (PCP 12, Hu Friedy, Chicago, USA) was inserted into the gingival sulcus, and bleeding was assessed dichotomously at six sites per tooth. The percentage of bleeding sites to measured sites was calculated. One trained and blinded investigator (AP) conducted all clinical assessments.

After computer-generated randomization undertaken by LS using Microsoft^®^ Office Excel, probands were allocated to either group A, which used the Oral-B^®^ iO™ Ultimate Clean brush head, or group B, which used the Oral-B^®^ Genius Cross-Action brush head. Calibrated study assistants instructed to the participants to ensure that the data collection remained blinded and unbiased for the examiner. Participants were instructed to refrain from interdental hygiene and use any chemical rinsing solution during the study period. Subjects of group A received hands-on training using the Oral-B^®^ iO™ with the Ultimate Clean brush head according to the manufacturer’s instructions. Subjects of the group B were asked to brush their teeth with the Oral-B^®^ Genius^®^ Cross-Action brush head and were also generously instructed. Both toothbrushes were held at a 90° angle to the tooth surface for five seconds on each tooth surface and then moved slowly along the gumline to follow the contour of the teeth. After detailed instruction, professional tooth cleaning was conducted with airflow polishing (*Airflow*^®^ prophylaxis master; Erythritol powder; EMS, Nyon, CH), and ultrasonic devices if necessary.

After completing the 28-day trial period using the assigned test products, the study participants presented for their second appointment. Their oral hygiene indices were re-examined, followed by a 14-day washout period, when the subjects resumed their normal cleaning routine. Participants were reassessed after 14 days (3rd visit). Again, oral hygiene indices were recorded, followed by a full plaque disclosure, thorough instruction for the second test product, and professional tooth cleaning. Analogous to the first test cycle, the study participants used the assigned toothbrush for 28 days before attending their fourth and final appointment for a hygiene index examination and professional tooth cleaning.

### Statistical analysis

Unless otherwise stated, median and interquartile range (IQR) are provided for descriptive analysis. On a proband level, RMNPI values were calculated as the total number of tooth areas with plaque present divided by the total number of tooth areas scored. This value corresponds to 28 teeth in a total of 504 sites for the whole mouth, 112 sites for the interdental areas, and 168 marginal area sites. An analogous calculation of the gingival bleeding index (GBI) was performed. The main outcome of whole-mouth RMNPI as well as GBI scores were compared between the two toothbrushing procedures using the Wilcoxon signed-rank test. The statistical analysis was conducted using IBM SPSS Statistics V.29.0.0.0 (IBM Armonk; NY, USA. The significance level was *p* < 0.05, with a power of 80%.

## Results

Seventeen women and 13 men with a mean age of 33.53 ± 7.53 years (range 20–66 years) finished the study (dropout rate 0%). This study analyzed 871 teeth.

### Plaque scores

**Fig. 3:**
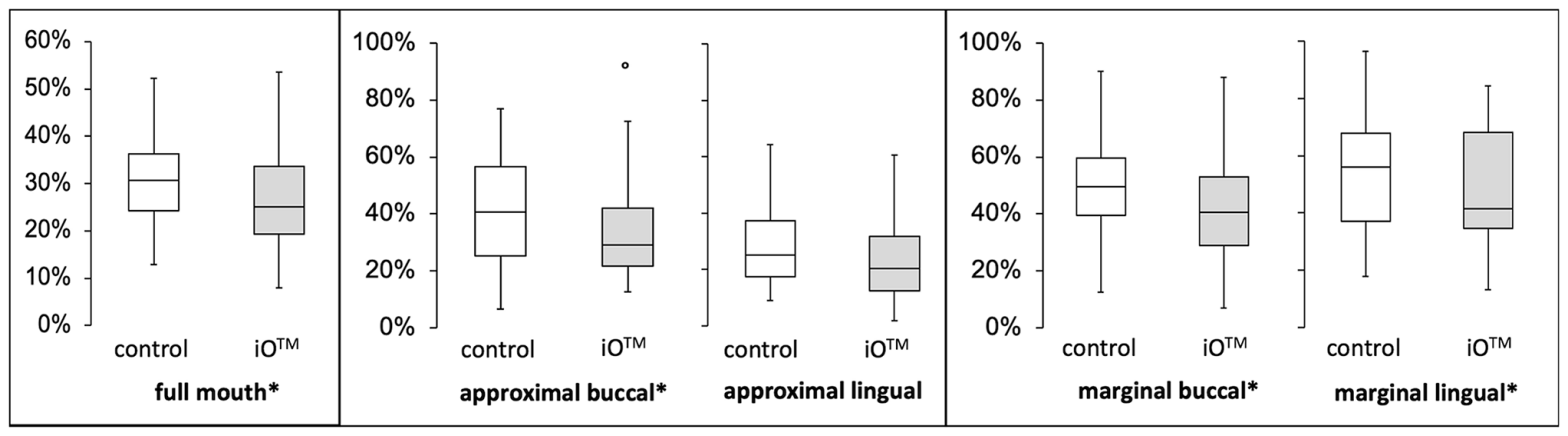
Rustogi Modified Navy Plaque Index after 28 days of home use. The Rustogi Modified Navy Plaque Index splits every buccal and lingual tooth surface into nine sections (A–I) and was calculated as a percentage of biofilm adhering sites to measured sites. The iO™ brush head showed statistically significantly lower plaque levels after 28 days of home use compared to the conventional oscillating-rotating toothbrush with the Cross-Action brush head (control) for full-mouth data (*p* = 0.019), approximal buccal sites (*p* < 0.001), marginal lingual/palatal (*p* < 0.001) and for marginal buccal sites (*p* = 0.027). The asterisk indicates statistically significant differences

At baseline, the median of the overall RMNPI was 40.05% (IQR 28.63–47.64).The two study groups had no statistically significant differences in baseline data. After 28 days of utilizing the iO™ brush head without interdental cleaning, the median overall RMNPI decreased to 25.09% (19.16–34.70). This value was statistically significantly lower than the median overall RMNPI attained after 28 days of using the Cross-Action brush head as a control procedure (median overall RMNPI 30.60%; IQR 23.45–36.39) (*p* = 0.029) (see Fig. 3).

Subgroup analysis revealed the higher cleansing efficiency of the Oral-B^®^ iO™ brush head was attributable to approximal and marginal sites (see Table 1). There was a statistically significantly lower plaque index after 28 days of cleaning with the iO™ compared to the Cross-Action on marginal lingual/palatal sites (median 36.85% versus 56.10%; *p* < 0.001) and on marginal buccal sites (median 40.24% versus 49.18%; *p* = 0.027). In addition, the iO™ exhibited a significantly lower plaque index than the Cross-action at approximal buccal sites (19.48% and 40.54%, respectively; *p* < 0.001). In contrast, no statistically significant difference existed in approximal lingual/palatal areas (see Table 1; Fig. 3).

**Table 1:** Plaque and bleeding indices after 28 days of home use. The Rustogi Modified Navy Plaque Index (RMNPI) split every buccal and lingual tooth surface into nine sections (A–I) and was calculated as a percentage of biofilm adhering sites to measured sites. The gingival bleeding index (GBI) was calculated dichotomously at six sites per tooth as a percentage of bleeding sites to measured sites. Data are presented using the median and interquartile range

	Oral-B^®^ iO	Oral-B^®^ Cross-Action	*p*-value
Full mouth			
RMNPI (%)	25.09 (19.16–34.70)	30.60 (23.45–36.39)	0.019
GBI (%)	0 (0–0.01)	0 (0–0.14)	0.904
Approximal sites			
RMNPI (%)	29.34 (17.64–37.58)	32.87 (25.0 − 45.13)	0.047
GBI (%)	0 (0–0.09)	0 (0–0.08)	0.517
Approximal buccal sites			
RMNPI (%)	19.48 (13.77–29.24)	40.54 (25.00–57.34)	< 0.001
GBI (%)	0 (0–0.04)	0	0.905
Approximal lingual / palatal sites			
RMNPI (%)	23.05 (11.92–33.54)	25.00 (16.91–39.71)	0.247
GBI (%)	0 (0–0.16)	0	0.675
Marginal sites			
RMNPI (%)	44.61 (31.34–58.63)	53.52 (37.11–62.68)	0.048
GBI (%)	0 (0–0.16)	0	0.396
Marginal buccal sites			
RMNPI (%)	40.24 (27.88–53.24)	49.18 (36.72–60.31)	0.027
GBI (%)	0	0 (0–0.33)	0.167
Marginal lingual / palatal sites			
RMNPI (%)	36.85 (27.99–51.04)	56.10 (34.35–68.42)	< 0.001
GBI (%)	0 (0–0.08)	0 (0–0.33)	0.858
Anterior Teeth			
RMNPI (%)	27.31 (19.44–37.96)	29.17 (19.68–43.29)	0.509
GBI (%)	0	0 (0–0.31)	0.064
Posterior Teeth			
RMNPI (%)	27.78 (20.66–34.86)	28.06 (21.53–35.42)	0.544
GBI (%)	0 (0–0.17)	0	0.985

RMNPI, Rustogi Modified Navy Plaque Index; GBI, gingival Bleeding Index; %, percent

### Gingival bleeding index

Statistical analysis revealed that unlike the plaque indices, gingival bleeding indices showed no statistically significant differences between the baseline and the test phase or between the different brush heads. At baseline, the median GBI was 1.54% (0–6.26). After using the iO™ for 28 days without any kind of interdental cleaning the median GBI was 0% (0–0.01). After 28 days of cleaning with the Cross-Action, the median GBI was 0% (0–0.14).

## Discussion

When new electric toothbrushes come onto the market, patients often ask whether they should invest in the new product, which is usually more expensive, or whether the previous one will not work just as well. One of the latest developments in the field of rotary-oscillating toothbrushes is the iO™ series by Oral-B^®^ with a magnetic drive system. The mechanism directs motor energy directly to the bristle tip [4]. The authors were primarily vexed by the dimensions of the iO™ brush heads, which boast a 2 mm larger diameter than one of its predecessors, the Cross-Action toothbrush head. Due to the brush head sizes, swiveling the brush head into the interdental spaces is no longer possible and is not recommended. Thus, the authors hypothesized that the larger brush head may clean the interdental spaces less effectively. Indeed, the null hypothesis was rejected due to a statistically significantly higher cleansing efficiency of the Oral-B^®^ iO™ toothbrush, mainly attributable to lower plaque levels at approximal and marginal sites (Table 1; Fig. 3).

The study compared the plaque reduction efficacy of two brush heads - the Ultimate Clean brush head from the iO™ series and the Cross-Action brush head with a toothbrush from the Oral-B^®^ Genius series - through a randomized-controlled and observer-blinded crossover methodology. A two-week washout phase was introduced between the two intervention cycles to avert a “carry-on” effect. Despite the increased risk of a carry-on effect when using a crossover study design, it shows some advantages over a parallel design [12]. Each contributor can serve as his own control, thereby decreasing the required sample size for the same statistical power. Intraoral conditions such as different restorative materials, existing teeth, and malocclusions such as crowding and crown molds influence plaque levels. However, the same intraoral conditions apply to both the test and the control product. All study participants were already using an oscillating-rotating electric toothbrush before entering the study, which reduced the risk of bias due to training over time. The test products were randomly and equally allocated to the study cycles to establish unbiased results further. Study participants received the necessary toothbrushing products and new brush heads before each cleaning cycle.

We could not calculate the sample size for this study as no previous studies had compared the new iO™ with conventional rotary-oscillating brushes. We therefore based our sample size on the requirements of the American Dental Association (ADA), which provides guidelines upon which toothbrushes can be considered for ADA acceptance. The ADA states that if a toothbrush does differ significantly in design or function from previously accepted toothbrushes, 30-day clinical trials of at least 30 participants per group will be required [13].

The plaque removal and gingival health effects of this new electric toothbrush technology were evaluated in randomized-controlled in-house studies of Oral-B^®^, including two 8-week trials versus a manual toothbrush and an 8-week and 6-month trial versus a sonic toothbrush [4, 6–8, 14]. The study results revealed statistically significantly greater plaque removal and better gum health benefits than those afforded by the Oral-B^®^ iO™ toothbrush technology over its manual and sonic toothbrush counterparts. For example, in the study by Grender et al., by week 8, more than 80% of the participants who used the Oral-B^®^ iO™ technology transitioned from “gingivitis” (having ≥ 10% bleeding sites) at the baseline to a “generally healthy” state (< 10% bleeding sites) [6]. In contrast, only 24% of participants in the manual toothbrush group and 53% in the sonic toothbrush group made similar transitions. Goyal et al. investigated the effects of the iO™ toothbrush and a sonic toothbrush on plaque reduction in a 6-month study [8]. The benefits of the experimental iO™ brush over the sonic brush were significantly greater in transitioning from “not healthy” to “healthy” gingivitis case status at week 24. By week 24, the O-R brush showed more plaque reduction in the whole mouth (24.6%), marginal (61.9%), and approximal regions 25.8% (p = 0.007) as opposed to the sonic brush (12.13%).

The current investigation is the first company-independent study evaluating the brushing efficacy of the iO™ series. After 28 days of home use without interdental cleaning and the use of any chemical rinsing solution, plaque control was notably more effective with the Ultimate Clean brush head and the iO™ toothbrush than with the conventional oscillating–rotating toothbrush and the Cross-Action brush head (RMNPI: Ultimate Clean 25.09% versus Cross-Action 30.60%, *p* = 0.019). This result was primarily attributable to marginal areas on both the buccal and lingual aspects of the teeth, as well as approximal surfaces on buccal sites. In contrast to prior studies, no difference in the gingival index appeared between the two products, probably due to the shorter study duration and the healthy gingival conditions at baseline. Studies by Deinzer and Ebel et al. have shown that even brushing to the best of one’s abilities with a manual toothbrush was not good enough, especially on marginal sites which showed persistent plaque at 69.48% ± 12.31% sites (mean ± SD) [15, 16]. Both the conventional O–R toothbrush and the iO™ toothbrush showed lower plaque levels here with 53.52% (37.11–62.68) and 44.61% (31.34–58.63) respectively (*p* < 0.001). The smooth surfaces are cleaned well with both brushes, which is also the case with manual toothbrushes, and the difference between the two brushes in the full-mouth RMNPI leveled out. However, the iO™ also had statistically significantly lower plaque values on the proximal buccal surfaces despite the larger brush head and the lack of twisting of the brush head into the interdental spaces (19.48% (range 13.77–29.24) and 40.54 (range 25–57.34), respectively, *p* = 0.027). The manufacturer claims that the new drive mechanism of the iO™ series creates microvibrations at the site of plaque removal, thus removing more plaque [4]. In our opinion, the slightly concave bristle field could also contribute to improved plaque reduction at marginal and approximal sites, as this shape of the brush head may adapt better to the shape of the teeth especially the molars.

In conclusion, the newly developed oscillating-rotating technology with the associated brush head exhibits more efficacy in reducing plaque than its predecessor. Additional comparative studies should be designed with parallel study designs and longer durations to substantiate the findings of this study.

## Data Availability

No datasets were generated or analysed during the current study.
